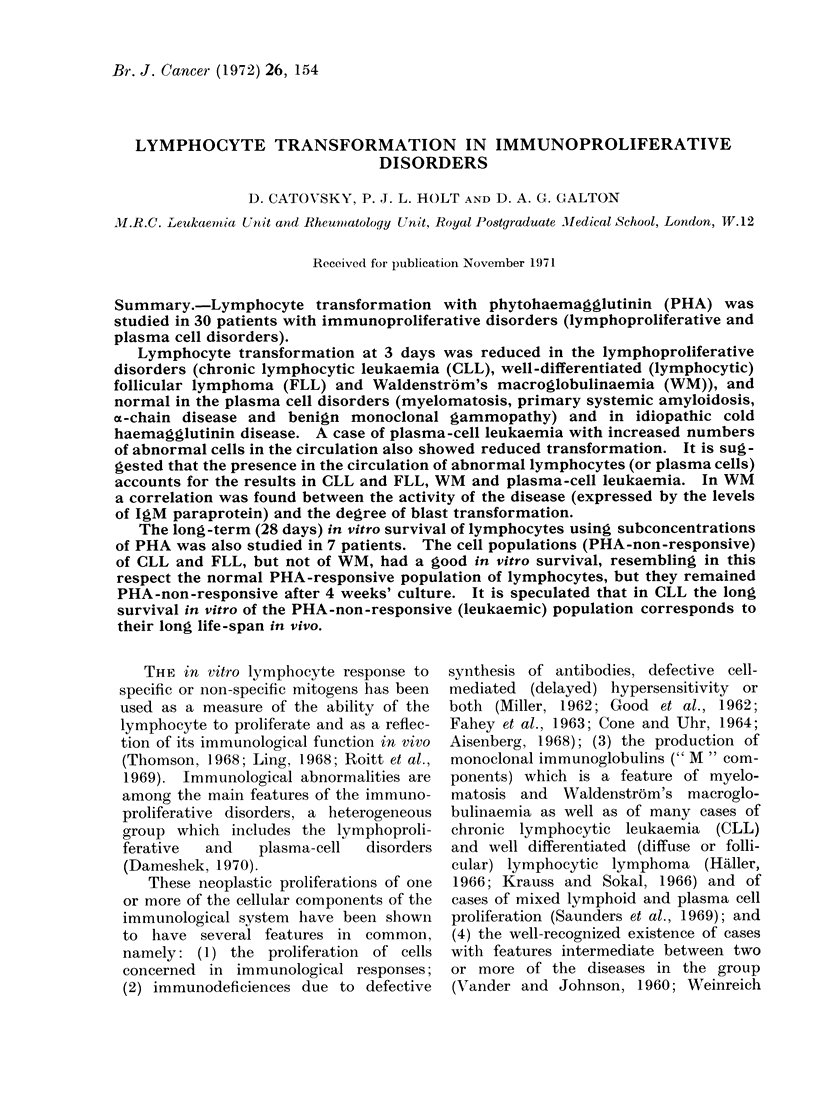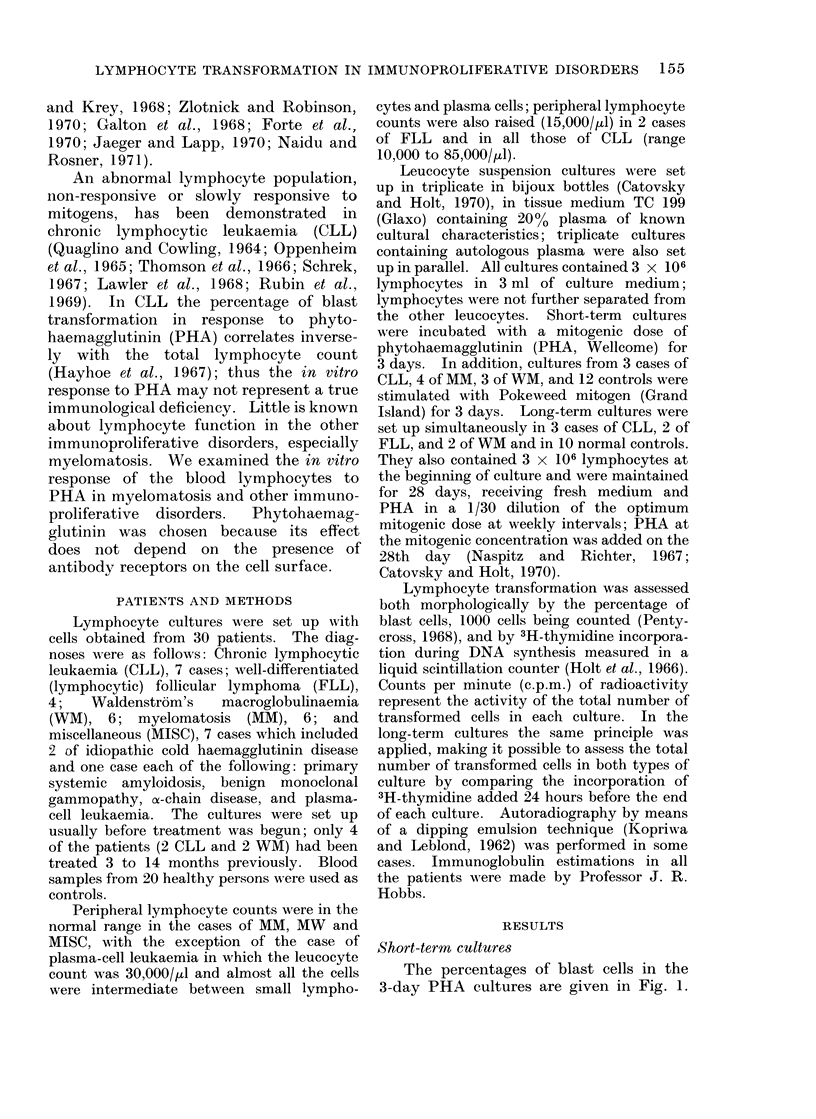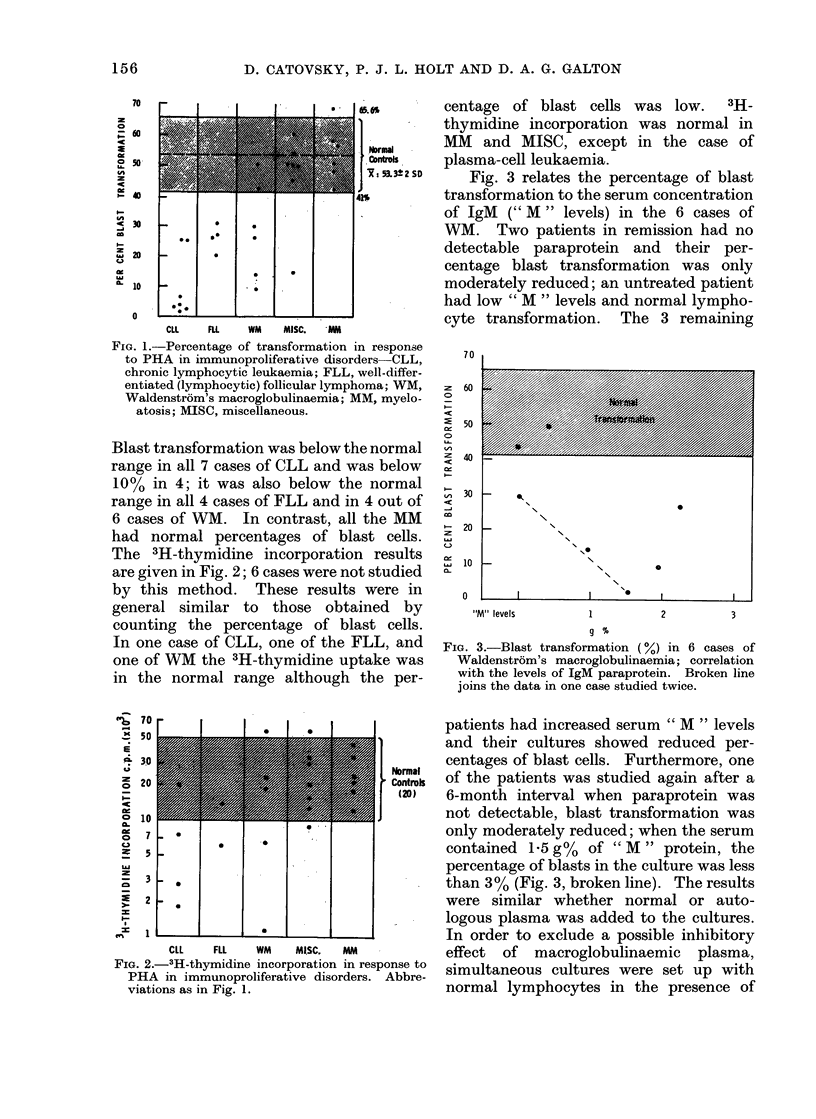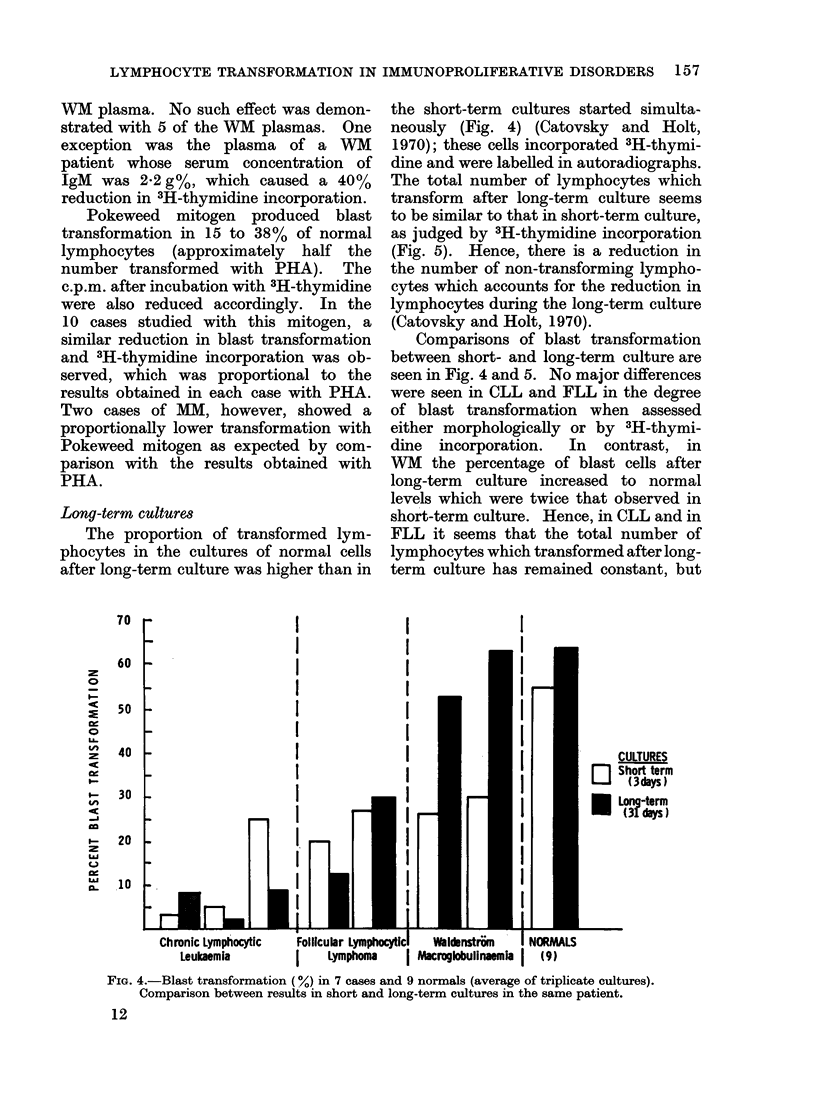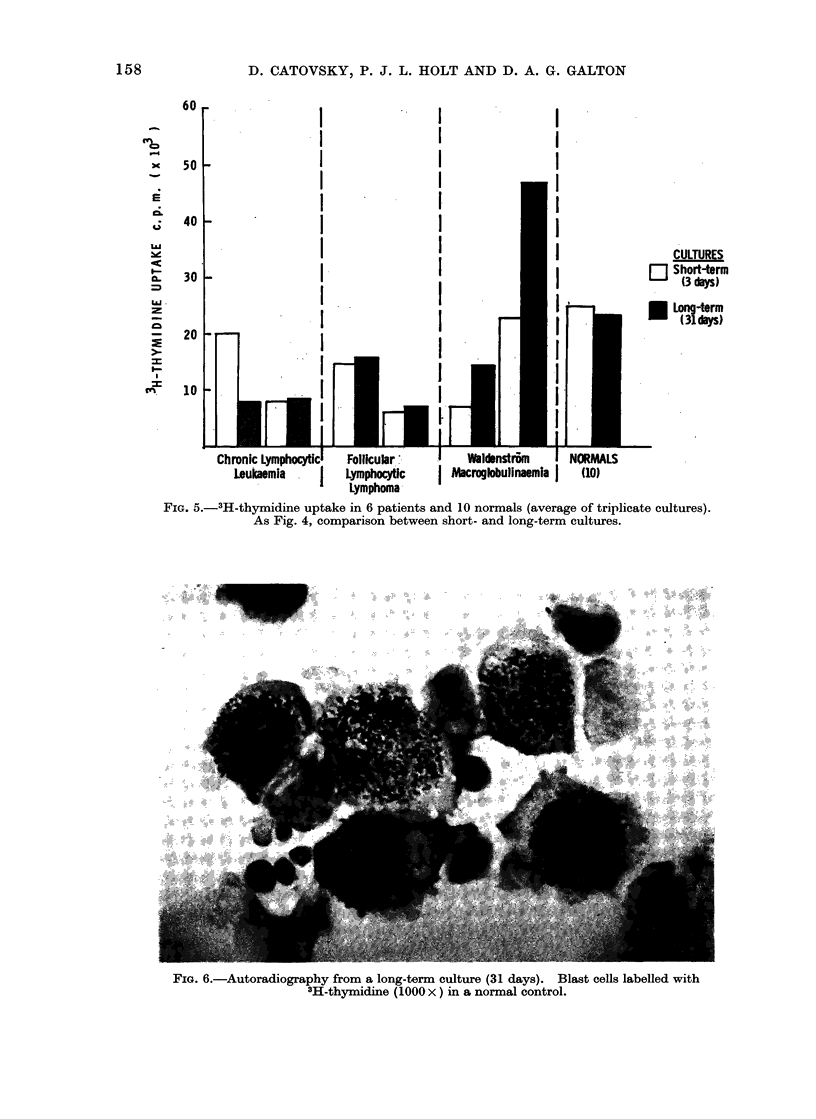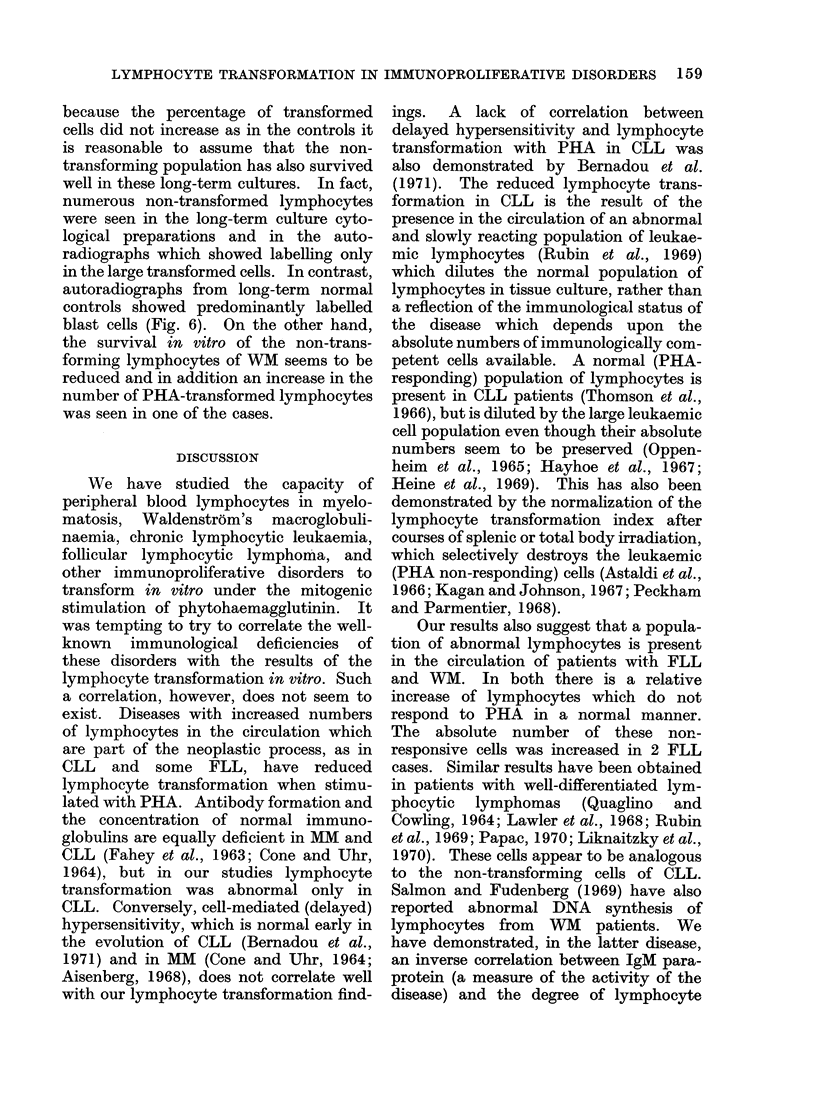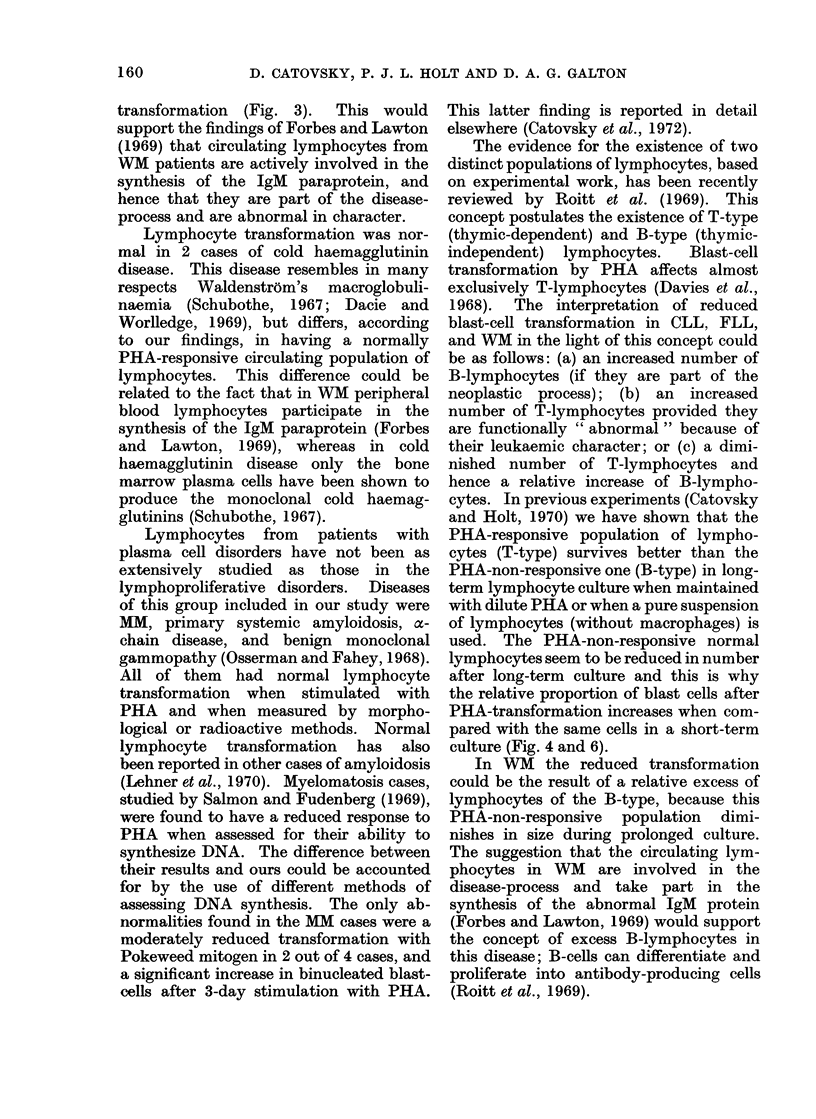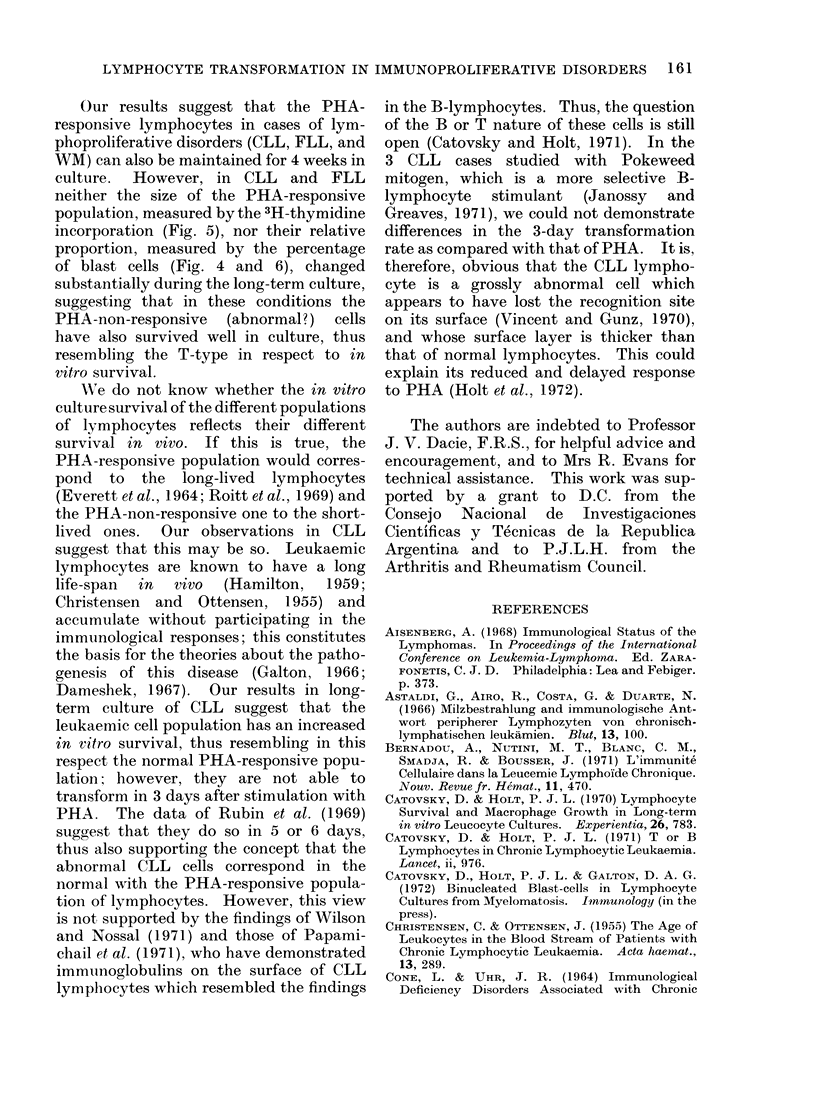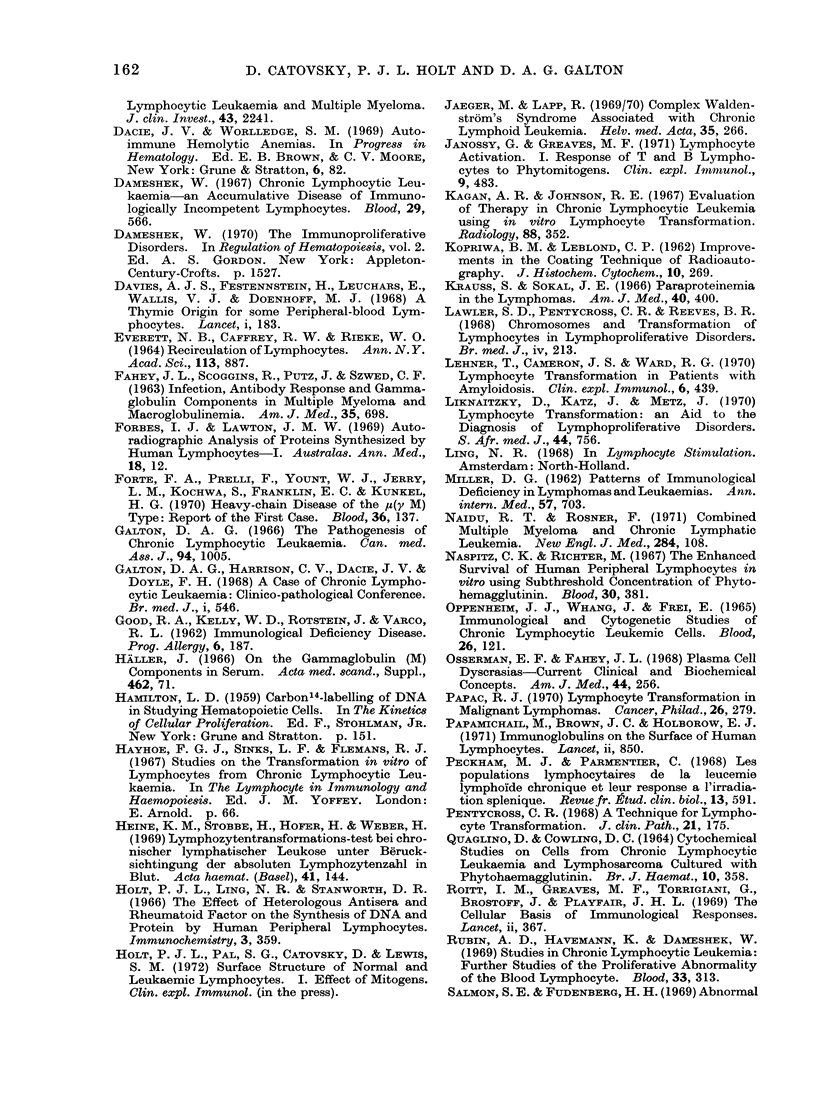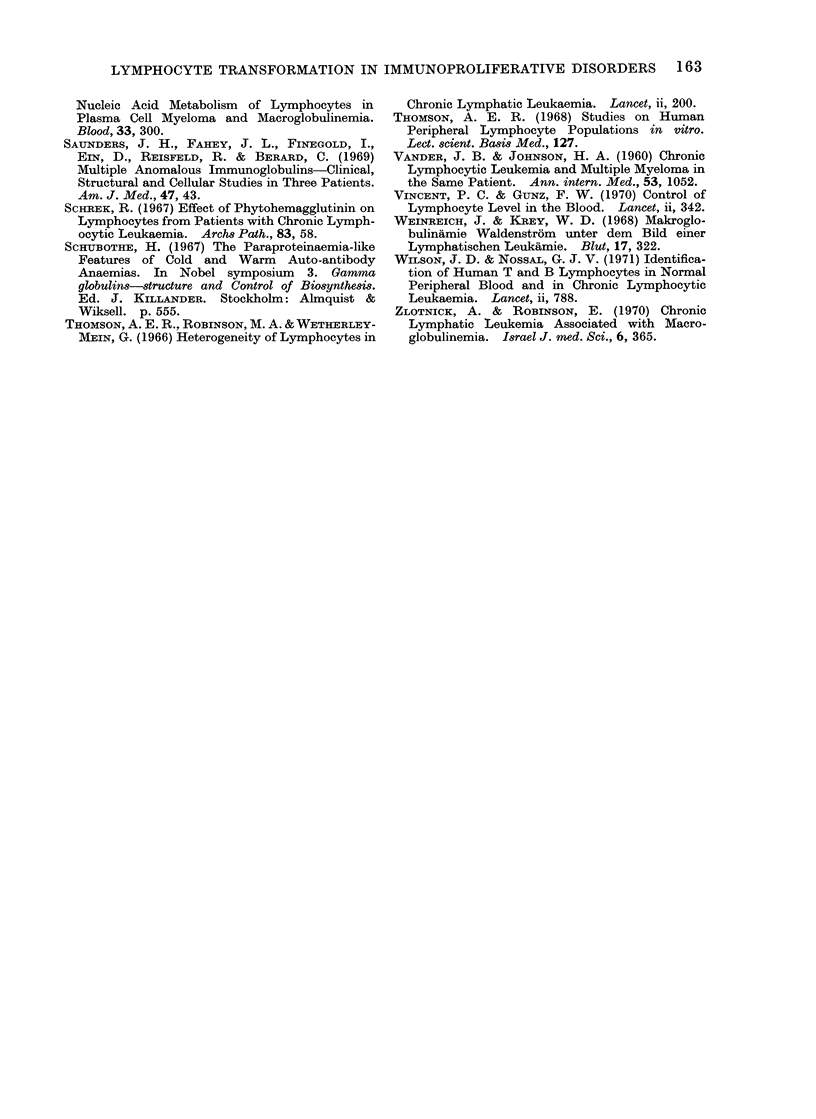# Lymphocyte Transformation in Immunoproliferative Disorders

**DOI:** 10.1038/bjc.1972.22

**Published:** 1972-06

**Authors:** D. Catovsky, P. J. L. Holt, D. A. G. Galton

## Abstract

**Images:**


*Br. J. Cancer* (1972) **26**, 154

### LYMPHOCYTE TRANSFORMATION IN IMMUNOPROLIFERATIVE DISORDERS

D. CATOVSKY, P. J. L. HOLT AND D. A. G. GALTON

*M.R.C. Leukaemia Unit and Rheumatology Unit, Royal Postgraduate Medical School, London, W.12*

Received for publication November 1971

**Summary.—Lymphocyte transformation with phytohaemagglutinin (PHA) was studied in 30 patients with immunoproliferative disorders (lymphoproliferative and plasma cell disorders).**

**Lymphocyte transformation at 3 days was reduced in the lymphoproliferative disorders (chronic lymphocytic leukaemia (CLL), well-differentiated (lymphocytic) follicular lymphoma (FLL) and Waldenström's macroglobulinaemia (WM)), and normal in the plasma cell disorders (myelomatosis, primary systemic amyloidosis, α-chain disease and benign monoclonal gammopathy) and in idiopathic cold haemagglutinin disease. A case of plasma-cell leukaemia with increased numbers of abnormal cells in the circulation also showed reduced transformation. It is suggested that the presence in the circulation of abnormal lymphocytes (or plasma cells) accounts for the results in CLL and FLL, WM and plasma-cell leukaemia. In WM a correlation was found between the activity of the disease (expressed by the levels of IgM paraprotein) and the degree of blast transformation.**

**The long-term (28 days) *in vitro* survival of lymphocytes using subconcentrations of PHA was also studied in 7 patients. The cell populations (PHA-non-responsive) of CLL and FLL, but not of WM, had a good *in vitro* survival, resembling in this respect the normal PHA-responsive population of lymphocytes, but they remained PHA-non-responsive after 4 weeks' culture. It is speculated that in CLL the long survival *in vitro* of the PHA-non-responsive (leukaemic) population corresponds to their long life-span *in vivo*.**

THE *in vitro* lymphocyte response to specific or non-specific mitogens has been used as a measure of the ability of the lymphocyte to proliferate and as a reflection of its immunological function *in vivo* (Thomson, 1968; Ling, 1968; Roitt *et al.*, 1969). Immunological abnormalities are among the main features of the immunoproliferative disorders, a heterogeneous group which includes the lymphoproliferative and plasma-cell disorders (Dameshek, 1970).

These neoplastic proliferations of one or more of the cellular components of the immunological system have been shown to have several features in common, namely: (1) the proliferation of cells concerned in immunological responses; (2) immunodeficiences due to defective synthesis of antibodies, defective cell-mediated (delayed) hypersensitivity or both (Miller, 1962; Good *et al.*, 1962; Fahey *et al.*, 1963; Cone and Uhr, 1964; Aisenberg, 1968); (3) the production of monoclonal immunoglobulins ("M" components) which is a feature of myelomatosis and Waldenström's macroglobulinaemia as well as of many cases of chronic lymphocytic leukaemia (CLL) and well differentiated (diffuse or follicular) lymphocytic lymphoma (Häller, 1966; Krauss and Sokal, 1966) and of cases of mixed lymphoid and plasma cell proliferation (Saunders *et al.*, 1969); and (4) the well-recognized existence of cases with features intermediate between two or more of the diseases in the group (Vander and Johnson, 1960; Weinreich and Krey, 1968; Zlotnick and Robinson, 1970; Galton *et al.*, 1968; Forte *et al.*, 1970; Jaeger and Lapp, 1970; Naidu and Rosner, 1971).

An abnormal lymphocyte population, non-responsive or slowly responsive to mitogens, has been demonstrated in chronic lymphocytic leukaemia (CLL) (Quaglino and Cowling, 1964; Oppenheim *et al.*, 1965; Thomson *et al.*, 1966; Schrek, 1967; Lawler *et al.*, 1968; Rubin *et al.*, 1969). In CLL the percentage of blast transformation in response to phytohaemagglutinin (PHA) correlates inversely with the total lymphocyte count (Hayhoe *et al.*, 1967); thus the *in vitro* response to PHA may not represent a true immunological deficiency. Little is known about lymphocyte function in the other immunoproliferative disorders, especially myelomatosis. We examined the *in vitro* response of the blood lymphocytes to PHA in myelomatosis and other immunoproliferative disorders. Phytohaemagglutinin was chosen because its effect does not depend on the presence of antibody receptors on the cell surface.

### PATIENTS AND METHODS

Lymphocyte cultures were set up with cells obtained from 30 patients. The diagnoses were as follows: Chronic lymphocytic leukaemia (CLL), 7 cases; well-differentiated (lymphocytic) follicular lymphoma (FLL), 4; Waldenström's macroglobulinaemia (WM), 6; myelomatosis (MM), 6; and miscellaneous (MISC), 7 cases which included 2 of idiopathic cold haemagglutinin disease and one case each of the following: primary systemic amyloidosis, benign monoclonal gammopathy, $\alpha$-chain disease, and plasma-cell leukaemia. The cultures were set up usually before treatment was begun; only 4 of the patients (2 CLL and 2 WM) had been treated 3 to 14 months previously. Blood samples from 20 healthy persons were used as controls.

Peripheral lymphocyte counts were in the normal range in the cases of MM, MW and MISC, with the exception of the case of plasma-cell leukaemia in which the leucocyte count was 30,000/$\mu$l and almost all the cells were intermediate between small lymphocytes and plasma cells; peripheral lymphocyte counts were also raised (15,000/$\mu$l) in 2 cases of FLL and in all those of CLL (range 10,000 to 85,000/$\mu$l).

Leucocyte suspension cultures were set up in triplicate in bijoux bottles (Catovsky and Holt, 1970), in tissue medium TC 199 (Glaxo) containing 20% plasma of known cultural characteristics; triplicate cultures containing autologous plasma were also set up in parallel. All cultures contained $3 \times 10^6$ lymphocytes in 3 ml of culture medium; lymphocytes were not further separated from the other leucocytes. Short-term cultures were incubated with a mitogenic dose of phytohaemagglutinin (PHA, Wellcome) for 3 days. In addition, cultures from 3 cases of CLL, 4 of MM, 3 of WM, and 12 controls were stimulated with Pokeweed mitogen (Grand Island) for 3 days. Long-term cultures were set up simultaneously in 3 cases of CLL, 2 of FLL, and 2 of WM and in 10 normal controls. They also contained $3 \times 10^6$ lymphocytes at the beginning of culture and were maintained for 28 days, receiving fresh medium and PHA in a 1/30 dilution of the optimum mitogenic dose at weekly intervals; PHA at the mitogenic concentration was added on the 28th day (Naspitz and Richter, 1967; Catovsky and Holt, 1970).

Lymphocyte transformation was assessed both morphologically by the percentage of blast cells, 1000 cells being counted (Pentycross, 1968), and by $^3$H-thymidine incorporation during DNA synthesis measured in a liquid scintillation counter (Holt *et al.*, 1966). Counts per minute (c.p.m.) of radioactivity represent the activity of the total number of transformed cells in each culture. In the long-term cultures the same principle was applied, making it possible to assess the total number of transformed cells in both types of culture by comparing the incorporation of $^3$H-thymidine added 24 hours before the end of each culture. Autoradiography by means of a dipping emulsion technique (Kopriwa and Leblond, 1962) was performed in some cases. Immunoglobulin estimations in all the patients were made by Professor J. R. Hobbs.

### RESULTS

### *Short-term cultures*

The percentages of blast cells in the 3-day PHA cultures are given in Fig. 1.

FIG. 1.—Percentage of transformation in response to PHA in immunoproliferative disorders—CLL, chronic lymphocytic leukaemia; FLL, well-differentiated (lymphocytic) follicular lymphoma; WM, Waldenström's macroglobulinaemia; MM, myeloatosis; MISC, miscellaneous.

Blast transformation was below the normal range in all 7 cases of CLL and was below 10% in 4; it was also below the normal range in all 4 cases of FLL and in 4 out of 6 cases of WM. In contrast, all the MM had normal percentages of blast cells. The $^{3}$H-thymidine incorporation results are given in Fig. 2; 6 cases were not studied by this method. These results were in general similar to those obtained by counting the percentage of blast cells. In one case of CLL, one of the FLL, and one of WM the $^{3}$H-thymidine uptake was in the normal range although the percentage of blast cells was low. $^{3}$H-thymidine incorporation was normal in MM and MISC, except in the case of plasma-cell leukaemia.

Fig. 3 relates the percentage of blast transformation to the serum concentration of IgM (" M " levels) in the 6 cases of WM. Two patients in remission had no detectable paraprotein and their percentage blast transformation was only moderately reduced; an untreated patient had low " M " levels and normal lymphocyte transformation. The 3 remaining

FIG. 3.—Blast transformation (%) in 6 cases of Waldenström's macroglobulinaemia; correlation with the levels of IgM paraprotein. Broken line joins the data in one case studied twice.

patients had increased serum " M " levels and their cultures showed reduced percentages of blast cells. Furthermore, one of the patients was studied again after a 6-month interval when paraprotein was not detectable, blast transformation was only moderately reduced; when the serum contained 1·5 g% of " M " protein, the percentage of blasts in the culture was less than 3% (Fig. 3, broken line). The results were similar whether normal or autologous plasma was added to the cultures. In order to exclude a possible inhibitory effect of macroglobulinaemic plasma, simultaneous cultures were set up with normal lymphocytes in the presence of

FIG. 2.—$^{3}$H-thymidine incorporation in response to PHA in immunoproliferative disorders. Abbreviations as in Fig. 1.

WM plasma. No such effect was demonstrated with 5 of the WM plasmas. One exception was the plasma of a WM patient whose serum concentration of IgM was 2·2 g%, which caused a 40% reduction in $^{3}$H-thymidine incorporation.

Pokeweed mitogen produced blast transformation in 15 to 38% of normal lymphocytes (approximately half the number transformed with PHA). The c.p.m. after incubation with $^{3}$H-thymidine were also reduced accordingly. In the 10 cases studied with this mitogen, a similar reduction in blast transformation and $^{3}$H-thymidine incorporation was observed, which was proportional to the results obtained in each case with PHA. Two cases of MM, however, showed a proportionally lower transformation with Pokeweed mitogen as expected by comparison with the results obtained with PHA.

*Long-term cultures*

The proportion of transformed lymphocytes in the cultures of normal cells after long-term culture was higher than in the short-term cultures started simultaneously (Fig. 4) (Catovsky and Holt, 1970); these cells incorporated $^{3}$H-thymidine and were labelled in autoradiographs. The total number of lymphocytes which transform after long-term culture seems to be similar to that in short-term culture, as judged by $^{3}$H-thymidine incorporation (Fig. 5). Hence, there is a reduction in the number of non-transforming lymphocytes which accounts for the reduction in lymphocytes during the long-term culture (Catovsky and Holt, 1970).

Comparisons of blast transformation between short- and long-term culture are seen in Fig. 4 and 5. No major differences were seen in CLL and FLL in the degree of blast transformation when assessed either morphologically or by $^{3}$H-thymidine incorporation. In contrast, in WM the percentage of blast cells after long-term culture increased to normal levels which were twice that observed in short-term culture. Hence, in CLL and in FLL it seems that the total number of lymphocytes which transformed after long-term culture has remained constant, but

FIG. 4.—Blast transformation (%) in 7 cases and 9 normals (average of triplicate cultures). Comparison between results in short and long-term cultures in the same patient.

FIG. 5.—$^3$H-thymidine uptake in 6 patients and 10 normals (average of triplicate cultures). As Fig. 4, comparison between short- and long-term cultures.

FIG. 6.—Autoradiography from a long-term culture (31 days). Blast cells labelled with $^3$H-thymidine (1000×) in a normal control.

because the percentage of transformed cells did not increase as in the controls it is reasonable to assume that the non-transforming population has also survived well in these long-term cultures. In fact, numerous non-transformed lymphocytes were seen in the long-term culture cytological preparations and in the autoradiographs which showed labelling only in the large transformed cells. In contrast, autoradiographs from long-term normal controls showed predominantly labelled blast cells (Fig. 6). On the other hand, the survival *in vitro* of the non-transforming lymphocytes of WM seems to be reduced and in addition an increase in the number of PHA-transformed lymphocytes was seen in one of the cases.

### DISCUSSION

We have studied the capacity of peripheral blood lymphocytes in myelomatosis, Waldenström's macroglobulinaemia, chronic lymphocytic leukaemia, follicular lymphocytic lymphoma, and other immunoproliferative disorders to transform *in vitro* under the mitogenic stimulation of phytohaemagglutinin. It was tempting to try to correlate the well-known immunological deficiencies of these disorders with the results of the lymphocyte transformation *in vitro*. Such a correlation, however, does not seem to exist. Diseases with increased numbers of lymphocytes in the circulation which are part of the neoplastic process, as in CLL and some FLL, have reduced lymphocyte transformation when stimulated with PHA. Antibody formation and the concentration of normal immunoglobulins are equally deficient in MM and CLL (Fahey *et al.*, 1963; Cone and Uhr, 1964), but in our studies lymphocyte transformation was abnormal only in CLL. Conversely, cell-mediated (delayed) hypersensitivity, which is normal early in the evolution of CLL (Bernadou *et al.*, 1971) and in MM (Cone and Uhr, 1964; Aisenberg, 1968), does not correlate well with our lymphocyte transformation findings. A lack of correlation between delayed hypersensitivity and lymphocyte transformation with PHA in CLL was also demonstrated by Bernadou *et al.* (1971). The reduced lymphocyte transformation in CLL is the result of the presence in the circulation of an abnormal and slowly reacting population of leukaemic lymphocytes (Rubin *et al.*, 1969) which dilutes the normal population of lymphocytes in tissue culture, rather than a reflection of the immunological status of the disease which depends upon the absolute numbers of immunologically competent cells available. A normal (PHA-responding) population of lymphocytes is present in CLL patients (Thomson *et al.*, 1966), but is diluted by the large leukaemic cell population even though their absolute numbers seem to be preserved (Oppenheim *et al.*, 1965; Hayhoe *et al.*, 1967; Heine *et al.*, 1969). This has also been demonstrated by the normalization of the lymphocyte transformation index after courses of splenic or total body irradiation, which selectively destroys the leukaemic (PHA non-responding) cells (Astaldi *et al.*, 1966; Kagan and Johnson, 1967; Peckham and Parmentier, 1968).

Our results also suggest that a population of abnormal lymphocytes is present in the circulation of patients with FLL and WM. In both there is a relative increase of lymphocytes which do not respond to PHA in a normal manner. The absolute number of these non-responsive cells was increased in 2 FLL cases. Similar results have been obtained in patients with well-differentiated lymphocytic lymphomas (Quaglino and Cowling, 1964; Lawler *et al.*, 1968; Rubin *et al.*, 1969; Papac, 1970; Liknaitzky *et al.*, 1970). These cells appear to be analogous to the non-transforming cells of CLL. Salmon and Fudenberg (1969) have also reported abnormal DNA synthesis of lymphocytes from WM patients. We have demonstrated, in the latter disease, an inverse correlation between IgM paraprotein (a measure of the activity of the disease) and the degree of lymphocyte transformation (Fig. 3). This would support the findings of Forbes and Lawton (1969) that circulating lymphocytes from WM patients are actively involved in the synthesis of the IgM paraprotein, and hence that they are part of the disease-process and are abnormal in character.

Lymphocyte transformation was normal in 2 cases of cold haemagglutinin disease. This disease resembles in many respects Waldenström's macroglobulinaemia (Schubothe, 1967; Dacie and Worlledge, 1969), but differs, according to our findings, in having a normally PHA-responsive circulating population of lymphocytes. This difference could be related to the fact that in WM peripheral blood lymphocytes participate in the synthesis of the IgM paraprotein (Forbes and Lawton, 1969), whereas in cold haemagglutinin disease only the bone marrow plasma cells have been shown to produce the monoclonal cold haemagglutinins (Schubothe, 1967).

Lymphocytes from patients with plasma cell disorders have not been as extensively studied as those in the lymphoproliferative disorders. Diseases of this group included in our study were MM, primary systemic amyloidosis, $\alpha$-chain disease, and benign monoclonal gammopathy (Osserman and Fahey, 1968). All of them had normal lymphocyte transformation when stimulated with PHA and when measured by morphological or radioactive methods. Normal lymphocyte transformation has also been reported in other cases of amyloidosis (Lehner *et al.*, 1970). Myelomatosis cases, studied by Salmon and Fudenberg (1969), were found to have a reduced response to PHA when assessed for their ability to synthesize DNA. The difference between their results and ours could be accounted for by the use of different methods of assessing DNA synthesis. The only abnormalities found in the MM cases were a moderately reduced transformation with Pokeweed mitogen in 2 out of 4 cases, and a significant increase in binucleated blast-cells after 3-day stimulation with PHA. This latter finding is reported in detail elsewhere (Catovsky *et al.*, 1972).

The evidence for the existence of two distinct populations of lymphocytes, based on experimental work, has been recently reviewed by Roitt *et al.* (1969). This concept postulates the existence of T-type (thymic-dependent) and B-type (thymic-independent) lymphocytes. Blast-cell transformation by PHA affects almost exclusively T-lymphocytes (Davies *et al.*, 1968). The interpretation of reduced blast-cell transformation in CLL, FLL, and WM in the light of this concept could be as follows: (a) an increased number of B-lymphocytes (if they are part of the neoplastic process); (b) an increased number of T-lymphocytes provided they are functionally " abnormal " because of their leukaemic character; or (c) a diminished number of T-lymphocytes and hence a relative increase of B-lymphocytes. In previous experiments (Catovsky and Holt, 1970) we have shown that the PHA-responsive population of lymphocytes (T-type) survives better than the PHA-non-responsive one (B-type) in long-term lymphocyte culture when maintained with dilute PHA or when a pure suspension of lymphocytes (without macrophages) is used. The PHA-non-responsive normal lymphocytes seem to be reduced in number after long-term culture and this is why the relative proportion of blast cells after PHA-transformation increases when compared with the same cells in a short-term culture (Fig. 4 and 6).

In WM the reduced transformation could be the result of a relative excess of lymphocytes of the B-type, because this PHA-non-responsive population diminishes in size during prolonged culture. The suggestion that the circulating lymphocytes in WM are involved in the disease-process and take part in the synthesis of the abnormal IgM protein (Forbes and Lawton, 1969) would support the concept of excess B-lymphocytes in this disease; B-cells can differentiate and proliferate into antibody-producing cells (Roitt *et al.*, 1969).

Our results suggest that the PHA-responsive lymphocytes in cases of lymphoproliferative disorders (CLL, FLL, and WM) can also be maintained for 4 weeks in culture. However, in CLL and FLL neither the size of the PHA-responsive population, measured by the $^{3}$H-thymidine incorporation (Fig. 5), nor their relative proportion, measured by the percentage of blast cells (Fig. 4 and 6), changed substantially during the long-term culture, suggesting that in these conditions the PHA-non-responsive (abnormal?) cells have also survived well in culture, thus resembling the T-type in respect to *in vitro* survival.

We do not know whether the *in vitro* culture survival of the different populations of lymphocytes reflects their different survival *in vivo*. If this is true, the PHA-responsive population would correspond to the long-lived lymphocytes (Everett *et al.*, 1964; Roitt *et al.*, 1969) and the PHA-non-responsive one to the short-lived ones. Our observations in CLL suggest that this may be so. Leukaemic lymphocytes are known to have a long life-span *in vivo* (Hamilton, 1959; Christensen and Ottensen, 1955) and accumulate without participating in the immunological responses; this constitutes the basis for the theories about the pathogenesis of this disease (Galton, 1966; Dameshek, 1967). Our results in long-term culture of CLL suggest that the leukaemic cell population has an increased *in vitro* survival, thus resembling in this respect the normal PHA-responsive population; however, they are not able to transform in 3 days after stimulation with PHA. The data of Rubin *et al.* (1969) suggest that they do so in 5 or 6 days, thus also supporting the concept that the abnormal CLL cells correspond in the normal with the PHA-responsive population of lymphocytes. However, this view is not supported by the findings of Wilson and Nossal (1971) and those of Papamichail *et al.* (1971), who have demonstrated immunoglobulins on the surface of CLL lymphocytes which resembled the findings in the B-lymphocytes. Thus, the question of the B or T nature of these cells is still open (Catovsky and Holt, 1971). In the 3 CLL cases studied with Pokeweed mitogen, which is a more selective B-lymphocyte stimulant (Janossy and Greaves, 1971), we could not demonstrate differences in the 3-day transformation rate as compared with that of PHA. It is, therefore, obvious that the CLL lymphocyte is a grossly abnormal cell which appears to have lost the recognition site on its surface (Vincent and Gunz, 1970), and whose surface layer is thicker than that of normal lymphocytes. This could explain its reduced and delayed response to PHA (Holt *et al.*, 1972).

The authors are indebted to Professor J. V. Dacie, F.R.S., for helpful advice and encouragement, and to Mrs R. Evans for technical assistance. This work was supported by a grant to D.C. from the Consejo Nacional de Investigaciones Científicas y Técnicas de la Republica Argentina and to P.J.L.H. from the Arthritis and Rheumatism Council.

### REFERENCES

AISENBERG, A. (1968) Immunological Status of the Lymphomas. In *Proceedings of the International Conference on Leukemia-Lymphoma*. Ed. ZARAFONETIS, C. J. D. Philadelphia: Lea and Febiger. p. 373.

ASTALDI, G., AIRO, R., COSTA, G. & DUARTE, N. (1966) Milzbestrahlung and immunologische Antwort peripherer Lymphozyten von chronischlymphatischen leukämien. *Blut*, **13**, 100.

BERNADOU, A., NUTINI, M. T., BLANC, C. M., SMADJA, R. & BOUSSER, J. (1971) L'immunité Cellulaire dans la Leucemie Lymphoïde Chronique. *Nouv. Revue fr. Hémat.*, **11**, 470.

CATOVSKY, D. & HOLT, P. J. L. (1970) Lymphocyte Survival and Macrophage Growth in Long-term *in vitro* Leucocyte Cultures. *Experientia*, **26**, 783.

CATOVSKY, D. & HOLT, P. J. L. (1971) T or B Lymphocytes in Chronic Lymphocytic Leukaemia. *Lancet*, ii, 976.

CATOVSKY, D., HOLT, P. J. L. & GALTON, D. A. G. (1972) Binucleated Blast-cells in Lymphocyte Cultures from Myelomatosis. *Immunology* (in the press).

CHRISTENSEN, C. & OTTENSEN, J. (1955) The Age of Leukocytes in the Blood Stream of Patients with Chronic Lymphocytic Leukaemia. *Acta haemat.*, **13**, 289.

CONE, L. & UHR, J. R. (1964) Immunological Deficiency Disorders Associated with Chronic

Lymphocytic Leukaemia and Multiple Myeloma. *J. clin. Invest.*, **43**, 2241.

DACIE, J. V. & WORLLEDGE, S. M. (1969) Autoimmune Hemolytic Anemias. In *Progress in Hematology*. Ed. E. B. BROWN, & C. V. MOORE, New York: Grune & Stratton, **6**, 82.

DAMESHEK, W. (1967) Chronic Lymphocytic Leukaemia—an Accumulative Disease of Immunologically Incompetent Lymphocytes. *Blood*, **29**, 566.

DAMESHEK, W. (1970) The Immunoproliferative Disorders. In *Regulation of Hematopoiesis*, vol. 2. Ed. A. S. GORDON. New York: Appleton-Century-Crofts. p. 1527.

DAVIES, A. J. S., FESTENNSTEIN, H., LEUCHARS, E., WALLIS, V. J. & DOENHOFF, M. J. (1968) A Thymic Origin for some Peripheral-blood Lymphocytes. *Lancet*, i, 183.

EVERETT, N. B., CAFFREY, R. W. & RIEKE, W. O. (1964) Recirculation of Lymphocytes. *Ann. N.Y. Acad. Sci.*, **113**, 887.

FAHEY, J. L., SCOGGINS, R., PUTZ, J. & SZWED, C. F. (1963) Infection, Antibody Response and Gammaglobulin Components in Multiple Myeloma and Macroglobulinemia. *Am. J. Med.*, **35**, 698.

FORBES, I. J. & LAWTON, J. M. W. (1969) Autoradiographic Analysis of Proteins Synthesized by Human Lymphocytes—I. *Australas. Ann. Med.*, **18**, 12.

FORTE, F. A., PRELLI, F., YOUNT, W. J., JERRY, L. M., KOCHWA, S., FRANKLIN, E. C. & KUNKEL, H. G. (1970) Heavy-chain Disease of the $\mu(\gamma$ M) Type: Report of the First Case. *Blood*, **36**, 137.

GALTON, D. A. G. (1966) The Pathogenesis of Chronic Lymphocytic Leukaemia. *Can. med. Ass. J.*, **94**, 1005.

GALTON, D. A. G., HARRISON, C. V., DACIE, J. V. & DOYLE, F. H. (1968) A Case of Chronic Lymphocytic Leukaemia: Clinico-pathological Conference. *Br. med. J.*, i, 546.

GOOD, R. A., KELLY, W. D., ROTSTEIN, J. & VARCO, R. L. (1962) Immunological Deficiency Disease. *Prog. Allergy*, **6**, 187.

HÄLLER, J. (1966) On the Gammaglobulin (M) Components in Serum. *Acta med. scand.*, Suppl., **462**, 71.

HAMILTON, L. D. (1959) Carbon$^{14}$-labelling of DNA in Studying Hematopoietic Cells. In *The Kinetics of Cellular Proliferation*. Ed. F., STOHLMAN, JR. New York: Grune and Stratton. p. 151.

HAYHOE, F. G. J., SINKS, L. F. & FLEMANS, R. J. (1967) Studies on the Transformation *in vitro* of Lymphocytes from Chronic Lymphocytic Leukaemia. In *The Lymphocyte in Immunology and Haemopoiesis*. Ed. J. M. YOFFEY. London: E. Arnold. p. 66.

HEINE, K. M., STOBBE, H., HOFER, H. & WEBER, H. (1969) Lymphozytentransformations-test bei chronischer lymphatischer Leukose unter Berücksichtingung der absoluten Lymphozytenzahl in Blut. *Acta haemat. (Basel)*, **41**, 144.

HOLT, P. J. L., LING, N. R. & STANWORTH, D. R. (1966) The Effect of Heterologous Antisera and Rheumatoid Factor on the Synthesis of DNA and Protein by Human Peripheral Lymphocytes. *Immunochemistry*, **3**, 359.

HOLT, P. J. L., PAL, S. G., CATOVSKY, D. & LEWIS, S. M. (1972) Surface Structure of Normal and Leukaemic Lymphocytes. I. Effect of Mitogens. *Clin. expl. Immunol.* (in the press).

JAEGER, M. & LAPP, R. (1969/70) Complex Waldenström's Syndrome Associated with Chronic Lymphoid Leukemia. *Helv. med. Acta*, **35**, 266.

JANOSSY, G. & GREAVES, M. F. (1971) Lymphocyte Activation. I. Response of T and B Lymphocytes to Phytomitogens. *Clin. expl. Immunol.*, **9**, 483.

KAGAN, A. R. & JOHNSON, R. E. (1967) Evaluation of Therapy in Chronic Lymphocytic Leukemia using *in vitro* Lymphocyte Transformation. *Radiology*, **88**, 352.

KOPRIWA, B. M. & LEBLOND, C. P. (1962) Improvements in the Coating Technique of Radioautography. *J. Histochem. Cytochem.*, **10**, 269.

KRAUSS, S. & SOKAL, J. E. (1966) Paraproteinemia in the Lymphomas. *Am. J. Med.*, **40**, 400.

LAWLER, S. D., PENTYCROSS, C. R. & REEVES, B. R. (1968) Chromosomes and Transformation of Lymphocytes in Lymphoproliferative Disorders. *Br. med. J.*, iv, 213.

LEHNER, T., CAMERON, J. S. & WARD, R. G. (1970) Lymphocyte Transformation in Patients with Amyloidosis. *Clin. expl. Immunol.*, **6**, 439.

LIKNAITZKY, D., KATZ, J. & METZ, J. (1970) Lymphocyte Transformation: an Aid to the Diagnosis of Lymphoproliferative Disorders. *S. Afr. med. J.*, **44**, 756.

LING, N. R. (1968) In *Lymphocyte Stimulation*. Amsterdam: North-Holland.

MILLER, D. G. (1962) Patterns of Immunological Deficiency in Lymphomas and Leukaemias. *Ann. intern. Med.*, **57**, 703.

NAIDU, R. T. & ROSNER, F. (1971) Combined Multiple Myeloma and Chronic Lymphatic Leukemia. *New Engl. J. Med.*, **284**, 108.

NASPITZ, C. K. & RICHTER, M. (1967) The Enhanced Survival of Human Peripheral Lymphocytes *in vitro* using Subthreshold Concentration of Phytohemagglutinin. *Blood*, **30**, 381.

OPPENHEIM, J. J., WHANG, J. & FREI, E. (1965) Immunological and Cytogenetic Studies of Chronic Lymphocytic Leukemic Cells. *Blood*, **26**, 121.

OSSERMAN, E. F. & FAHEY, J. L. (1968) Plasma Cell Dyscrasias—Current Clinical and Biochemical Concepts. *Am. J. Med.*, **44**, 256.

PAPAC, R. J. (1970) Lymphocyte Transformation in Malignant Lymphomas. *Cancer, Philad.*, **26**, 279.

PAPAMICHAIL, M., BROWN, J. C. & HOLBOROW, E. J. (1971) Immunoglobulins on the Surface of Human Lymphocytes. *Lancet*, ii, 850.

PECKHAM, M. J. & PARMENTIER, C. (1968) Les populations lymphocytaires de la leucemie lymphoïde chronique et leur response a l'irradiation splenique. *Revue fr. Étud. clin. biol.*, **13**, 591.

PENTYCROSS, C. R. (1968) A Technique for Lymphocyte Transformation. *J. clin. Path.*, **21**, 175.

QUAGLINO, D. & COWLING, D. C. (1964) Cytochemical Studies on Cells from Chronic Lymphocytic Leukaemia and Lymphosarcoma Cultured with Phytohaemagglutinin. *Br. J. Haemat.*, **10**, 358.

ROITT, I. M., GREAVES, M. F., TORRIGIANI, G., BROSTOFF, J. & PLAYFAIR, J. H. L. (1969) The Cellular Basis of Immunological Responses. *Lancet*, ii, 367.

RUBIN, A. D., HAVEMANN, K. & DAMESHEK, W. (1969) Studies in Chronic Lymphocytic Leukemia: Further Studies of the Proliferative Abnormality of the Blood Lymphocyte. *Blood*, **33**, 313.

SALMON, S. E. & FUDENBERG, H. H. (1969) Abnormal

Nucleic Acid Metabolism of Lymphocytes in Plasma Cell Myeloma and Macroglobulinemia. *Blood*, **33**, 300.

SAUNDERS, J. H., FAHEY, J. L., FINEGOLD, I., EIN, D., REISFELD, R. & BERARD, C. (1969) Multiple Anomalous Immunoglobulins—Clinical, Structural and Cellular Studies in Three Patients. *Am. J. Med.*, **47**, 43.

SCHREK, R. (1967) Effect of Phytohemagglutinin on Lymphocytes from Patients with Chronic Lymphocytic Leukaemia. *Archs Path.*, **83**, 58.

SCHUBOTHE, H. (1967) The Paraproteinaemia-like Features of Cold and Warm Auto-antibody Anaemias. In Nobel symposium 3. *Gamma globulins—structure and Control of Biosynthesis.* Ed. J. KILLANDER. Stockholm: Almquist & Wiksell. p. 555.

THOMSON, A. E. R., ROBINSON, M. A. & WETHERLEY-MEIN, G. (1966) Heterogeneity of Lymphocytes in Chronic Lymphatic Leukaemia. *Lancet*, ii, 200.

THOMSON, A. E. R. (1968) Studies on Human Peripheral Lymphocyte Populations *in vitro*. *Lect. scient. Basis Med.*, **127**.

VANDER, J. B. & JOHNSON, H. A. (1960) Chronic Lymphocytic Leukemia and Multiple Myeloma in the Same Patient. *Ann. intern. Med.*, **53**, 1052.

VINCENT, P. C. & GUNZ, F. W. (1970) Control of Lymphocyte Level in the Blood. *Lancet*, ii, 342.

WEINREICH, J. & KREY, W. D. (1968) Makroglobulinämie Waldenström unter dem Bild einer Lymphatischen Leukämie. *Blut*, **17**, 322.

WILSON, J. D. & NOSSAL, G. J. V. (1971) Identification of Human T and B Lymphocytes in Normal Peripheral Blood and in Chronic Lymphocytic Leukaemia. *Lancet*, ii, 788.

ZLOTNICK, A. & ROBINSON, E. (1970) Chronic Lymphatic Leukemia Associated with Macroglobulinemia. *Israel J. med. Sci.*, **6**, 365.